# MicroRNA-mRNA Regulatory Networking Fine-Tunes Polyunsaturated Fatty Acid Synthesis and Metabolism in the Inner Mongolia Cashmere Goat

**DOI:** 10.3389/fgene.2021.649015

**Published:** 2021-06-02

**Authors:** Yuchun Xie, Zhihong Liu, Juntao Guo, Xin Su, Cun Zhao, Chongyan Zhang, Qing Qin, Dongliang Dai, Yanhong Zhao, Zhiying Wang, Ruijun Wang, Yanjun Zhang, Rui Su, Zhixin Wang, Jinquan Li

**Affiliations:** ^1^College of Animal Science, Inner Mongolia Agricultural University, Hohhot, China; ^2^Key Laboratory of Animal Genetics, Breeding and Reproduction, Hohhot, China; ^3^Key Laboratory of Mutton Sheep Genetics and Breeding, Ministry of Agriculture, Hohhot, China; ^4^Engineering Research Center for Goat Genetics and Breeding, Hohhot, China

**Keywords:** microRNA, mRNA, cashmere goat, ACSL1, fatty acid

## Abstract

Fatty acid composition is an important aspect of meat quality in ruminants. Improving the beneficial fatty acid level in cashmere goat meat is important to its economic value. To investigate microRNAs (miRNAs) and mRNAs that regulate or coregulate polyunsaturated fatty acid (PUFA) synthesis and metabolism in the Inner Mongolia cashmere goat, we used longissimus dorsi muscle (WLM) and biceps femoris muscle (WBM) for transcript-level sequencing. RT-qPCR was used to evaluate the expression of mRNAs and miRNAs associated with PUFA synthesis and metabolism. The total PUFA content in the WBM was significantly higher than that in the WLM (*P* < 0.05). Our study is the first to systematically report miRNAs in cashmere goat meat. At the mRNA level, 20,375 genes were identified. *ACSL1*, *CD36* and *TECRL* were at the center of a gene regulatory network and contributed significantly to the accumulation and metabolic regulation of fatty acids. At the miRNA level, 426 known miRNAs and 30 novel miRNAs were identified. KEGG analysis revealed that the miRNA target genes were involved mainly in the PPAR signaling pathway. The mRNA-miRNA coregulation analysis showed that *ACSL1* was negatively targeted by nine miRNAs: chi-miR-10a-5p, chi-miR-10b-5p, chi-miR-130b-5p, chi-miR-15a-5p_R-1, chi-miR-15b-5p, chi-miR-16a-5p, chi-miR-16b-5p, chi-miR-181c-5p_R+1, and chi-miR-26b-5p. Finally, we speculated that the simultaneous silencing of *ACSL1* by one or more of these nine miRNAs through PPAR signaling led to low *ACSL1* expression in the WLM and, ultimately to high PUFA content in the WBM. Our study helps elucidate the metabolic regulation of fatty acids in Inner Mongolia cashmere goats.

## Introduction

The Inner Mongolian cashmere goat is a local breed that provides both cashmere and meat, but it is famous for its cashmere, which is known as “soft gold” (Su et al., 2015, 2020). The cashmere value of this goat has often caused its meat value to be ignored. Every year, nearly 700,000 cashmere goats are maintained, and the corresponding meat production is nearly 10,000 tons. Although the cashmere of this goat has been widely studied, its meat economic traits have not been reported. Fatty acid composition is a crucial aspect of meat quality in ruminants, as fatty acids often influence the flavor and juiciness of meat (Tvrzicka et al., 2011). In addition, fatty acids in meat are important for human health (Ladeira et al., 2018). Improving the quality of meat by increasing the concentrations of beneficial fatty acids to improve human health and reducing those of potentially detrimental fatty acids is an important undertaking (Scollan et al., 2014).

In the past few years, an increasing number of studies have demonstrated that microRNAs (miRNAs), once considered genomic “noise,” can mediate the epigenetic, transcriptional, and posttranscriptional regulation of genes involved in fatty acid metabolism by participating in the fatty acid regulatory network (Xu et al., 2010; Long et al., 2019; Zhang et al., 2019). Such studies have linked fatty acid metabolism with miRNA-mRNA relationships, which may provide new ideas and directions for exploration of the regulatory mechanisms of fatty acid metabolism in animals. Lu et al. (2020) found that miR-212 regulates fatty acid synthase (*FASN*) and sterol regulatory element binding factor 1 (*SREBP1*) expression by targeting the silent information regulator 2 (*SIRT2*) gene and repressing its expression, ultimately promoting fat deposition in mammary epithelial cells. In intramuscular preadipocytes, overexpression of miR-17-5p suppresses the expression of nuclear receptor coactivator 3 (*NCOA3*), fatty acid binding protein 4 (*FABP4*), and peroxisome proliferator-activated receptor gamma (PPARγ) and inhibits the differentiation of preadipocytes (Han et al., 2017). Depending on the degree of complementarity between miRNAs and the 3′-untranslated region (UTR) or 5′-UTR sequences of target mRNAs, miRNAs regulate mRNAs in two ways (Bartel, 2009): If the sequences are fully complementary, the miRNAs degrade the target mRNAs, whereas if the sequences are incompletely complementary, the miRNAs repress mRNA translation and thereby protein synthesis. Both ways accomplish gene regulation. Ma et al. (2018) found that overexpression of bta-miR-130a/b inhibits the expression of adipocyte differentiation-related genes, including peroxisome proliferator activated receptor gamma (*PPARG)*, fatty acid binding protein 4 (*FABP4)*, lipin 1 (*LPIN1)*, and lipoprotein lipase (*LPL*). The aim of this study was to investigate polyunsaturated fatty acids (PUFAs) that are differentially present in different parts of the same goat, e.g., in the longissimus dorsi muscle (WLM) and biceps femoris muscle (WBM), and to use these two muscles as models to screen key miRNAs and mRNAs related to fatty acid synthesis and metabolism at the transcriptional level. Joint analysis of RNAs at both the mRNA and miRNA levels was further performed to construct a key miRNA-mRNA network associated with fatty acid metabolism and synthesis, which was further validated. The findings of this study help lay a foundation for the elucidation of the metabolic regulation of fatty acids in cashmere goats.

## Materials and Methods

### Ethics Statement

Samples were collected in accordance with the Guidelines for Experimental Animals of the Ministry of Science and Technology (Beijing, China) and were approved by the experimental animal ethics committee of Inner Mongolia Agricultural University (Approval No. [2020]056).

### Animals

The animals were slaughtered under controlled conditions after being electrically stunned. WLM and WBM tissues were excised immediately between the 12th and 13th ribs (right half carcass) and rapidly stored in liquid nitrogen and subsequently stored at –80°C until analysis. Three replicates were provided by Inner Mongolia cashmere goats (Yiwei White Cashmere Goat Breeding Farm, Erdos, Inner Mongolia). In semidesert pastures, all goats are grazed year-round and feed freely. Two muscles from three goats (25.2 ± 0.16 kg, wether, 2 years old) were used for fatty acid composition analysis, miRNA sequencing (miRNA-Seq) and mRNA sequencing (mRNA-Seq) and RT-qPCR.

### Fatty Acid Composition Analysis

The extracted fatty acids were prepared with fatty acid methyl esters (Berton et al., 2016). The fatty acids were separated by an Agilent 5977B GC/MSD (Agilent, CA, United States) using a 100 m × 0.25 mm diameter SP-2560 capillary column of 0.02 mm thickness (Supelco, Bellefonte, PA) (Ebrahimi et al., 2014). Helium was used as a carrier gas with a spit ratio of 9:1. The experimental conditions for mass spectrometry were as follows: full scan mode, a solvent delay of 11 min, a gain factor of 10, an ion source temperature of 230°C, and a quadrupole temperature of 150°C. Qualitative Analysis B.07.00 software (Agilent) was used with the National Institute of Standards and Technology (NIST) database to identify the compound information for the reference standard (Sigma-Aldrich, United States) and samples. The results are expressed as a percentage of total fatty acids.

### Total RNA Isolation, miRNA-Seq and mRNA-Seq

Total RNA was extracted using TRIzol reagent (Invitrogen, CA, United States) following the manufacturer’s procedure. The 6000 Nano LabChip Kit (Agilent, CA, United States) with the Agilent Bioanalyzer 2100 system (Agilent Technologies, CA, United States) was used to assess RNA integrity. Approximately 1 μg of total RNA with an RNA integrity number (RIN) > 7.0 was used to prepare a small RNA library according to the protocol of a TruSeq Small RNA Sample Prep Kit (Illumina, San Diego, United States). Then, we performed single-end sequencing (36 bp or 50 bp) on an Illumina HiSeq 2500. Approximately 10 μg of total RNA representing a specific adipose type was used to deplete ribosomal RNA with an Epicenter Ribo-Zero Gold Kit (Illumina). Following purification, the poly(A)- or poly(A)+ RNA fractions were fragmented into small pieces using divalent cations under elevated temperature. Then, the cleaved RNA fragments were reverse-transcribed to create the final cDNA library in accordance with the protocol for a mRNA-Seq sample preparation kit (Illumina), and the average insert size for the paired-end libraries was 300 bp (±50 bp). Then, we performed paired-end sequencing on an Illumina HiSeq 4000 (lc-bio, China) following the vendor’s recommended protocol.

### Raw Data Analysis for mRNAs and miRNAs

First, Cutadapt (Martin, 2011) was used to remove the reads that contained adaptor sequences, contamination, low-quality bases and undetermined bases. Then, sequence quality was verified with FastQC^1^. We used Bowtie2 (Langmead and Salzberg, 2012) and TopHat2 (Kim et al., 2013) to map reads to the genome of *Capra hircus*. The mapped reads of each sample were assembled with StringTie (Pertea et al., 2015). Subsequently, the whole transcriptome of *Capra hircus* was obtained. The samples were merged to reconstruct a comprehensive transcriptome using Perl scripts. After the final transcriptome was generated, StringTie and Ballgown (Frazee et al., 2015) were used to estimate the expression levels of all transcripts.

The raw reads were subjected to an in-house program, ACGT101-miR (LC Sciences, Houston, Texas, United States), to remove adapter dimers, contamination, low-complexity sequences, sequences of common RNA families (rRNA, tRNA, small nuclear RNA (snRNA), snoRNA) and repeats. Subsequently, unique sequences with lengths of 18--26 nucleotides were mapped to specific species precursors in miRBase 22.0 with the Basic Local Alignment Search Tool (BLAST) to identify known miRNAs and novel 3p- and 5p-derived miRNAs. Length variation at both the 3′ and 5′ ends and one mismatch within the sequence were allowed in the alignment. The unique sequences that mapped to the hairpin arms of mature species-specific miRNAs were identified as known miRNAs. The unique sequences that mapped to the arms of known species-specific precursors opposite the annotated mature miRNA-containing arm were considered to be novel 5p- or 3p-derived miRNA candidates. The remaining sequences were mapped to precursors from other selected species (excluding species-specific precursors) in miRBase 22.0 through BLAST search, and the mapped premiRNAs were further BLASTed against the specific species genomes to determine their genomic locations. We defined the miRNAs obtained through the above two methods as known miRNAs. The unmapped sequences were BLASTed against the specific genomes, and the hairpin RNA structures containing sequences were predicted from the flanking 80 nt sequences using RNAfold software^2^.

### Differential Expression Analysis of mRNAs and miRNAs Related to Fatty Acid Synthesis and Metabolism

StringTie was used to determine mRNA expression levels by calculating the fragments per kilobase of transcript per million mapped reads (FPKM) values. The differentially expressed mRNAs meeting the criteria of a log2 (fold change, FC) > 1 or a log2 (FC) < –1 and statistical significance (*P* < *0.05*) were selected with the R package Ballgown.

The differential expression of miRNAs based on normalized deep-sequencing counts was analyzed through Student’s *t*-test based on the experimental design. The significance thresholds were 0.01 and 0.05 in each test. For the prediction of the target genes of the most abundant miRNAs, two computational target prediction algorithms (TargetScan 5.0 and miRanda 3.3a) were used to identify miRNA binding sites. When using the miRanda database, the species of Capra hircus was used. Finally, the genes predicted by both algorithms were combined, and the overlapping genes were identified. The Gene Ontology (GO) terms and Kyoto Encyclopedia of Genes and Genomes (KEGG) pathways of the most abundant miRNAs and their targets were also annotated.

### Validation of the RNA-Seq of mRNAs and miRNAs Related to Fatty Acid Synthesis and Metabolism

Total RNA from six samples (three goats) was extracted with TRIzol (Invitrogen, CA) according to the manufacturer’s instructions. Total RNA was reverse-transcribed to cDNA using the PrimeScript RT reagent Kit (TaKaRa, Tokyo, Japan) and RT-qPCR was performed using SYBR Premix Ex Taq (TaKaRa, Tokyo, Japan). The mRNA primers were synthesized by Shanghai Shenggong, China (Supplementary Table 9). The relative mRNA levels were normalized to β-actin (Hou et al., 2016) expression for each sample; For miRNA detection, reverse transcription followed by stem-loop RT-qPCR was performed according to the manufacturer’s protocols using the Bulge-LoopTM miRNA RT-qPCR Primer (RiboBio, Guangzhou, China). Quantification of miRNA was performed with a stem-loop real-time PCR miRNA kit (RiboBio). The relative miRNA levels were normalized to U6 small nuclear RNA (Carvalho et al., 2019) expression for each sample.

### Statistical Analysis for RT-qPCR of mRNA and miRNA

The ct data from three goats and three technical replicates of all the candidate mRNA and miRNA obtained from the RT-qPCR experiments were evaluated with the 2^–ΔΔ*CT*^ method (Livak and Schmittgen, 2001). Student’s *t*-test was performed for WBM and WLM using SPSS (IBM SPSS Statistics). The results are presented as the mean ± standard deviation. A significance level of 0.05 was used.

## Results

### Fatty Acid Content of WLM and WBM

**FIGURE 1:**
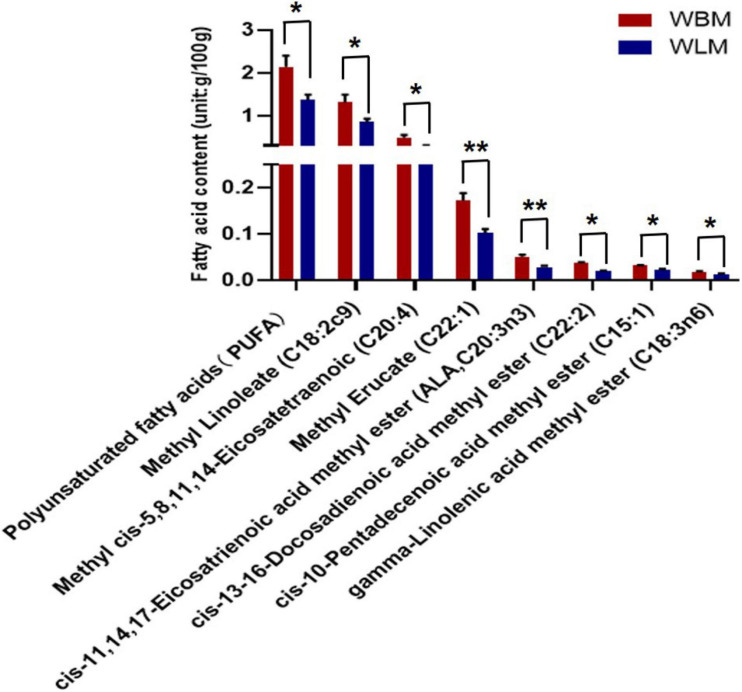
Fatty acid content of longissimus dorsi muscle (WLM) and biceps femoris muscle (WBM). Red represents the high-fatty acid group (the WBM tissue group), while blue represents the low-fatty acid group (the WLM tissue group). The asterisks represent the levels of significance (*t*-test: *P < 0.05, **P < 0.01).

To assess the fatty acid content, WLM and WBM tissues were collected from 2-year-old goats. A total of 34 fatty acids were found in the muscles of Inner Mongolian cashmere goats, including the omega-3 fatty acids C22:6 and C18:3n3 and the omega-6 fatty acids C18:2c9, C18:3n6, and C20:4 (Supplementary Table 1). Independent-sample *t*-tests were performed for fatty acids in different muscles (WBM and WLM). The results showed that the total PUFA content in WBM was significantly higher than that in WLM (*P* < *0.05*) (Figure 1 and Supplementary Table 2), and the levels of the PUFAs methyl linoleate (C18:2c9), cis-13-16-docosadienoic acid methyl ester (C22:2), gamma-linolenic acid methyl ester (C18:3n6), cis-11,14,17-eicosatrienoic acid methyl ester (C20:3n3), methyl cis-5,8,11,11, and 14-eicosatetraenoic acid (C20:4) in WBM were significantly higher than those in WLM (*P* < *0.05*). The levels of the monounsaturated fatty acids (MUFAs) cis-10-pentadecenoic acid methyl ester (C15:1) and methyl erucate (C22:1) in WBM were significantly higher than those in WLM (*P* < *0.05*). In other words, WBM and WLM were representative and could be used as models for the study of PUFAs.

### The Expression of mRNAs and miRNAs in Different Muscles of Inner Mongolia Cashmere Goats

To assess the mRNAs and miRNAs of the Inner Mongolia cashmere goat, we collected WLM tissues and WBM tissues for transcriptomic profiling of all mRNAs and miRNAs via high-throughput sequencing. For RNA-Seq library preparation, an average of 88,125,888 clean reads were obtained from the six samples tested, and 82.23–85.22% of these reads were uniquely aligned to the reference genome ARS1 in Ensembl. All 6 samples had at least 95.41% reads with quality equal to or exceeding Q30. An average of 20,375 genes were found in each sample of Inner Mongolia cashmere goat meat (Supplementary Table 3).

In addition, for the small RNA-Seq libraries, an average of 11,226,053 clean reads and 190,214 unique reads were obtained. An average of 426 known miRNAs and 30 novel miRNAs were obtained after a series of analyses (Supplementary Table 4).

### Differentially Expressed Genes (DEGs) Related to Unsaturated Fatty Acid Synthesis and Metabolism

**FIGURE 2:**
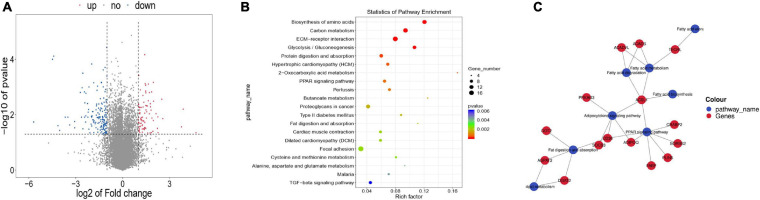
Differentially expressed genes (DEGs) in skeletal muscle in the high-fatty acid group and the low-fatty acid group. **(A)** DEGs. The red and blue dots represent upregulated and downregulated mRNAs, respectively. **(B)** KEGG pathway analysis for the DEGs. Only the top 20 enriched pathways are presented here. **(C)** KEGG-DEGs pairs and regulatory network.

There were 245 DEGs in WBM compared with WLM(Figure 2A and Supplementary Table 5), of which 85 were upregulated and 160 were downregulated in WBM. These DEGs included 18 DEGs related to the regulation of fatty acid synthesis and metabolism, including acyl-CoA synthetase long chain family member 1 (*ACSL1*) (FC = 2.23), lactate dehydrogenase B (*LDHB*, FC = 3.44), acyl-CoA dehydrogenase (*ACADS*, FC = 2.02), long-chain fatty acid acyl-CoA dehydrogenase (*ACADVL*, FC = 2.56), and trans-2,3-enoyl-CoA reductase like (*TECRL*) (FC = 2.67). These genes are important for the accumulation and metabolism of PUFAs.

KEGG analysis revealed pathways associated with these DEGs (Figure 2B and Supplementary Table 6). Specifically, the DEGs were involved in 8 fatty acid-related signaling pathways, including the fat digestion and absorption (ko04975), fatty acid biosynthesis (ko00061), fatty acid metabolism (ko01212), fatty acid elongation (ko00062), PPAR signaling (ko03320), adipocytokine (ko04920) pathways, fatty acid degradation (ko00071), and glycerolipid metabolism(ko00561) pathways (Table 1). These eight signaling pathways are also important for the accumulation and metabolism of PUFAs. The mRNA-based KEGG network analysis of the above 18 DEGs and 8 signaling pathways revealed an indirect relationship among the 15 DEGs (Figure 2C). *ACSL1* was associated with 5 fatty acid metabolic process terms: the adipocytokine signaling pathway, fatty acid biosynthesis, fatty acid metabolism, fatty acid degradation, and PPAR signaling pathway were thus indirectly linked to 11 genes. *CD36* was found to participate in fat digestion and absorption, the PPAR signaling pathway and the adipocytokine signaling pathway and was indirectly linked to 11 genes. *TECRL* was revealed to be involved in fatty acid metabolism, fatty acid elongation and the biosynthesis of PUFAs and was indirectly linked to three genes. *ACSL1*, *CD36* and *TECRL* were at the center of the gene regulatory network, indicating that they contribute significantly to the accumulation and metabolic regulation of fatty acids.

**TABLE 1:** Eight significantly enriched pathways related to fatty acid metabolism.

**Pathway_id**	**Pathway_name**	**Genes**	**DEG number**	***P*-value**
ko00062	Fatty acid elongation	*TECRL*	1	0.364156
ko00561	Glycerolipid metabolism	*AGPAT2*; *DGAT2*	2	0.292918
ko00061	Fatty acid biosynthesis	*ACSL1*	1	0.249221
ko00071	Fatty acid degradation	*ACADS*; *ACADVL*; *ACSL1*	3	0.073432
ko01212	Fatty acid metabolism	*ACADS*; *ACADVL*; *ACSL1*; *TECRL*	4	0.022678
ko04920	Adipocytokine signaling pathway	*ACSL1*; *ADIPOQ*; *CD36*; *PRKAG3*; *SOCS3*	5	0.019141
ko03320	PPAR signaling pathway	*ACSL1*; *ADIPOQ*; *CD36*; *CRABP2*; *FABP*; *PLIN5*; *SORBS2*	7	0.00259
ko04975	Fat digestion and absorption	*AGPAT2*; *CD36*; *DGAT2*; *GOT2*	4	0.002178

### Differentially Expressed miRNAs (DEMs) Related to Unsaturated Fatty Acid Synthesis and Metabolism

**FIGURE 3:**
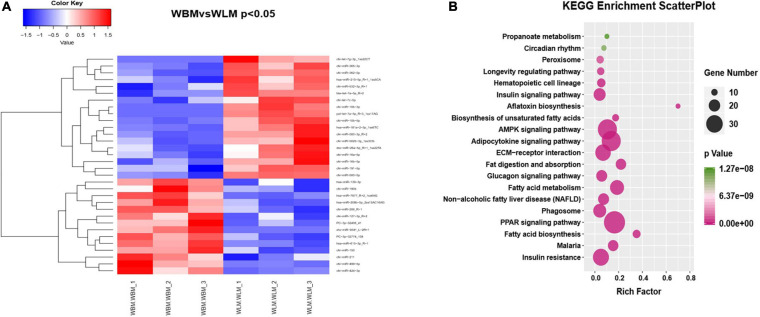
Differentially expressed miRNAs (DEMs) in skeletal muscle in the high-fatty acid group and the low-fatty acid group. **(A)** Heatmap of the significant DEMs. Red represents significant upregulation, while blue represents significant downregulation. **(B)** KEGG analysis of the targets of DEMs related to fatty acid metabolism.

Venn analysis of the 409 miRNAs found in cashmere goat muscle showed that a total of 343 miRNAs were co-expressed in both WBM and WLM, 39 were expressed only in WLM, and 27 were expressed only in WBM (Supplementary Table 7). A total of 32 (*P* < 0.05) DEMs were found in different parts of the muscle (Figure 3A and Supplementary Table 8), of which 14 were upregulated and 18 were downregulated in WBM. PC-3p-32774_108 and hsa-miR-615-3p_R-1 were upregulated (*P* < 0.01), whereas chi-miR-10b-3p, pol-let-7a-5p_R+3_1ss17AG, chi-miR-362-5p, and bta-let-7a-5p_R+2 were downregulated (*P* < 0.01).

KEGG analysis of the miRNA target genes (Figure 3B and Supplementary Table 8) revealed that they were associated mainly with biological processes such as fatty acid biosynthesis, the PPAR signaling pathway, and fatty acid degradation and absorption. The miRNA functional analysis results were consistent with the KEGG analysis results for the DEGs.

**FIGURE 4:**
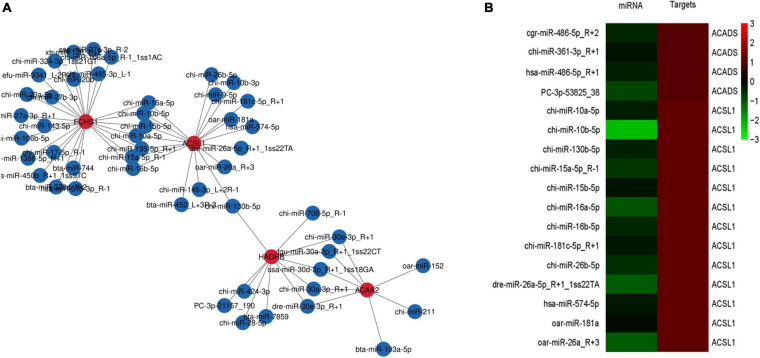
mRNA-microRNA coregulation analysis **(A)**. miRNA-mRNA coregulatory network during fatty acid metabolism. The red and blue circles represent mRNAs and miRNAs, respectively. The solid lines indicate coregulation between miRNAs and mRNAs. **(B)** Clustering analysis of DEGs and miRNA expression patterns.

The miRNA target genes were predicted, and the transcriptome data were then used as a bridge to integrate the two sets of association data to obtain a miRNA-mRNA coregulatory network (Figure 4A). The constructed network contained 4 DEGs and 57 miRNAs. The network revealed that acetyl-CoA acyltransferase 2 (*ACAA2*) could be targeted by 8 miRNAs, while hydroxyacyl-CoA dehydrogenase trifunctional multienzyme complex subunit beta (*HADHB*) could be targeted by 11 miRNAs. *ACSL1* could be targeted by 15 miRNAs, and enoyl-CoA hydratase short chain 1 (*ECHS1*) could be targeted by 23 miRNAs. These results suggest that *ACAA2*, *HADHB*, *ACSL1*, and *ECHS1* may be the crucial genes mediated by miRNAs for the regulation of fatty acid metabolism.

To determine which specific miRNAs regulate the DEGs related to fatty acid metabolism, we performed clustering analysis of the expression patterns of the target genes (Figure 4B). The analysis revealed the existence of 9 miRNAs that negatively regulate the *ACSL1* gene in cashmere goats, including chi-miR-10a-5p, chi-miR-10b-5p, chi-miR-130b-5p, chi-miR-15a-5p_R-1, chi-miR-15b-5p, chi-miR-16a-5p, chi-miR-16b-5p, chi-miR-181c-5p_R + 1, and chi-miR-26b-5p. chi-miR-361-3p negatively regulates the *ACADS* gene in cashmere goats.

### mRNA and miRNA Expression Related to Unsaturated Fatty Acid Synthesis and Metabolism

**FIGURE 5:**
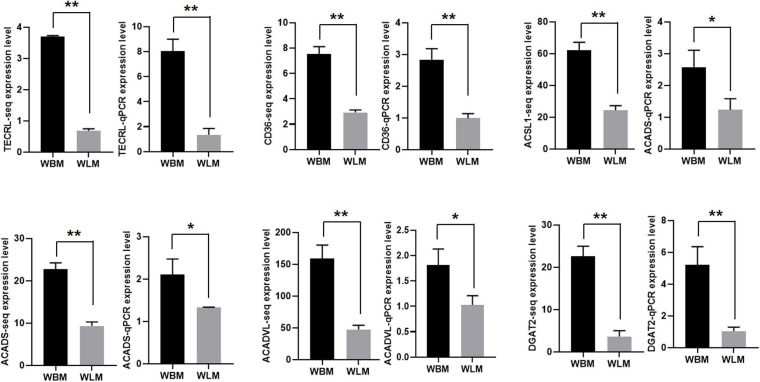
The expression of mRNA related to unsaturated fatty acid synthesis and metabolism. Biceps femoris muscle (WBM): high-fatty acid content group. Longissimus dorsi muscle (WLM): low-fatty acid content group. Expression of genes were normalized by β-actin. The asterisks represent the levels of significance (*t*-test: *P < 0.05, **P < 0.01).

The expression of 6 DEGs related to unsaturated fatty acid synthesis and metabolism [*TECRL*, *CD36*, *ACSL1*, *ACADS*, *ACADVL* and diacylglycerol o-acyltransferase 2 (*DGAT2*)] was validated through RT-qPCR (Figure 5). The expression of these selected transcripts was significantly higher in the WBM than in the WLM tissues (*P* < 0.05), and the expression patterns were highly consistent with those obtained by the RNA-Seq method.

**FIGURE 6:**
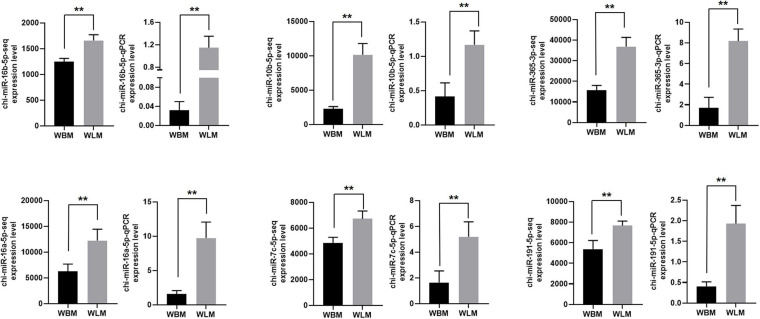
Validation of the miRNA-Seq results. Biceps femoris muscle (WBM): high-fatty acid group. Longissimus dorsi muscle (WLM): low-fatty acid group. Expression of miRNAs were normalized by U6 snRNA. The asterisks represent the levels of significance (*t*-test: **P < 0.01).

The RNA-Seq results for chi-miR-16b-5p, chi-miR-10b-5p, chi-miR-365-3p, chi-miR-16a-5p, chi-miR-7c-5p, and chi-miR-191-5p were also validated through RT-qPCR (Figure 6). The expression of these selected miRNAs was significantly higher in the WLM than in the WBM tissues (*P* < 0.01), and the expression patterns were highly consistent with those obtained by the RNA-Seq method.

## Discussion

Our results show that Inner Mongolian cashmere goat meat contains a variety of omega-3 and omega-6 fatty acids that are beneficial to humans, such as C18:3n3 and C18:2c9, which have protective effects against cardiovascular and inflammatory diseases via the downregulation of proinflammatory genes (De Caterina et al., 2006; Yalcintan et al., 2018). There are two essential types of fatty acids in human nutrition: omega-3 fatty acids (alpha-linolenic acid) and omega-6 fatty acids (linoleic acid). The human body cannot synthesize essential fatty acids and thus must obtain them from food. Although seafood is the major dietary source of omega-3 fatty acids, a recent fatty acid intake survey indicated that red meat also serves as a significant source of omega-3 fatty acids for some populations (Sinclair et al., 1994). Therefore, understanding the molecular mechanisms related to fatty acids in the meat of the Inner Mongolian cashmere goat is vital. Improving our understanding of the sensory characteristics and nutritional value of goat meat is also key. Fatty acid composition is species- and tissue-specific. In our study, the PUFA content was significantly higher in WBM than in WLM. The findings indicate that the WBM and WLM of Inner Mongolia cashmere goats are effective models for research on the accumulation and metabolism of PUFAs.

Improving the beneficial fatty acid content of cashmere goat meat is important to improving the economic traits of cashmere goats. Thus, it is necessary to understand the molecules and molecular mechanisms that affect the accumulation and metabolism of fatty acids. To investigate which genes and miRNAs coregulate PUFA expression in Inner Mongolia cashmere goats, we selected WBM and WLM tissues with significantly different PUFA contents for miRNA and mRNA sequencing. We identified 20,375 genes and 426 known miRNAs and 30 novel miRNAs in the meat of Inner Mongolia cashmere goats. At the mRNA level, 18 DEGs and 8 signaling pathways were related to fatty acid metabolism. The KEGG network analysis of the above 18 DEGs and 8 signaling pathways revealed that *ACSL1*, *CD36*, and *TECRL* are at the center of the gene regulatory network. *ACSL1* exists in fat cells and is considered to play an important role in activating the synthesis of triglycerides from fatty acids (Li et al., 2009). High expression of *ACSL1* reduces fatty acid β-oxidation through the PPARγ pathway, thereby increasing triglyceride levels (Li T. et al., 2020). In addition, *CD36* can activate fatty acid β-oxidation (Shi and Burn, 2004; Kennedy and Kashyap, 2011). *CD36^+^CD44*^bright^ cells express relatively high levels of three key enzymes involved in fatty acid β-oxidation (*ACADVL*, *ACADM*, and *HADHA*) (Pascual et al., 2017). *CD36* recognizes a number of lipid ligands, binds native and oxidized lipoproteins and then coordinates fat metabolism (Pepino et al., 2014). Furthermore, through GeneCards, we found that *TECRL* is a protein-coding gene. Among its related pathways are metabolism and fatty acyl-CoA biosynthesis. However, there have been no reports about *TECRL* in muscle tissue. In conclusion, the *ACSL1*, *CD36*, and *TECRL* genes are important participants in molecular mechanisms related to fatty acids in the Inner Mongolia cashmere goat.

At the miRNA level, our study is the first to systematically report that miRNAs regulate PUFA metabolism in the meat of Inner Mongolia cashmere goats. There were 214, 222, and 207 mature miRNAs in skeletal muscles of Landrace, Tongcheng, and Wuzhishan pigs, respectively (Hou et al., 2016). There were 767 known miRNAs in the WLM of Xinjiang brown cattle and Kazakh cattle (Li N. et al., 2020). In our study, we identified 426 known miRNAs and 30 novel miRNAs in the meat of cashmere goats. The number of miRNAs identified in the meat of cashmere goats was less than that in beef, but more than that in pork. Our results provide data to elucidate the molecular mechanisms affecting PUFA metabolism in Inner Mongolia cashmere goats. KEGG analysis showed that the miRNA target genes were mainly involved in fatty acid biosynthesis, fatty acid elongation, fatty acid degradation and absorption, and the PPAR signaling pathway. These results are consistent with those of the DEG KEGG analysis. Fatty acid uptake and decreased lipolysis are associated with increased intramuscular fat (IMF) deposition. Specifically, changes in the balance between synthesis and degradation can increase or decrease fatty acids (Teixeira et al., 2017). PPARs are a family of nuclear receptors that bind to fatty acids and perform significant functions in the regulation of nutrient metabolism and energy homeostasis (Lemay and Hwang, 2006). PUFAs can bind to PPARα at physiological concentrations and induce the expression of several genes involved in fatty acid metabolism, including fatty acid transport, synthesis and β oxidation (Rodríguez-Cruz and Serna, 2017). In conclusion, the PPAR pathway is important for fatty acid metabolism in the Inner Mongolia cashmere goat.

The mRNA-miRNA coregulation analysis showed that only *ACSL1* was negatively regulated by miRNAs in cashmere goats. *ACSL1* was negatively targeted by 9 miRNAs: chi-miR-10a-5p, chi-miR-10b-5p, chi-miR-130b-5p, chi-miR-15a-5p_R-1, chi-miR-15b-5p, chi-miR-16a-5p, chi-miR-16b-5p, chi-miR-181c-5p_R + 1, and chi-miR-26b-5p. *ACSL1* is involved in 5 fatty acid metabolic pathways: the adipocytokine signaling pathway, fatty acid biosynthesis, fatty acid metabolism, fatty acid degradation, and the PPAR signaling pathway; all pathways are related to PPAR signaling. Thus, we speculated that the simultaneous silencing of *ACSL1* by one or more of these nine negatively regulated miRNAs through PPAR signaling led to the low expression of the *ACSL1* gene in the WLM and finally to the high PUFA content in the WBM. A study by Lian showed that bta-miR-181a may contribute to the negative regulation of lipid synthesis in mammary cells by targeting *ACSL1* (Lian et al., 2016). In addition, miR-34a-5p can increase intracellular lipid content by reducing the *ACSL1* protein level (Tian et al., 2019). miR-126-3 might be involved in lipid metabolism in the mammary gland (Chu et al., 2017). However, there were no prior reports on the 9 miRNAs identified in our study and their association with fatty acids in cashmere goat meat. Furthermore, which miRNAs interact with *ACSL1* through PPAR signaling pathways to ultimately affect fatty acid synthesis or breakdown in the Inner Mongolia cashmere goat requires further cellular functional validation.

## Conclusion

Our study is the first to systematically report miRNAs in cashmere goat meat and 426 known miRNAs and 30 novel miRNAs were identified. KEGG analysis revealed that the miRNA target genes were involved mainly in the PPAR signaling pathway. At the mRNA level, 20375 genes were identified. The mRNA-miRNA coregulation analysis showed that *ACSL1* was negatively targeted by nine miRNAs. We speculated that the simultaneous silencing of *ACSL1* by one or more of these nine miRNAs through PPAR signaling led to low *ACSL1* expression in the WLM and, ultimately, to high PUFA content in the WBM.

## Data Availability Statement

The data presented in the study are deposited in the SRA database (https://www.ncbi.nlm.nih.gov/sra) repository, accession number (SRA, PRJNA689238).

## Ethics Statement

The animal study was reviewed and approved by the experimental animal ethics committee of Inner Mongolia Agricultural University [Approval No. (2020)056].

## Author Contributions

YX, ZL, and JG made substantial contributions to the conception and design of the experiments. YX, ZL, JG, XS, CYZ, and CZ conceived and designed the experiments YX, RS, QQ, and DD performed the experiments. YX, ZL, ZYW, YJZ, and RW analyzed the data. YX, ZL, YHZ, and ZYW wrote the manuscript. JL and ZXW critically revised the manuscript. All authors read and approved the final manuscript.

## Conflict of Interest

The authors declare that the research was conducted in the absence of any commercial or financial relationships that could be construed as a potential conflict of interest.

## Footnotes

1 http://www.bioinformatics.babraham.ac.uk/projects/fastqc/

2 http://rna.tbi.univie.ac.at/cgi-bin/RNAfold.cgi

## Supplementary Material

Supplementary Table 1 Fatty acid content of cashmere goat muscle.

Supplementary Table 2 Fatty acid classification.

Supplementary Table 3 Overview of the data for RNA sequencing.

Supplementary Table 4 Overview of the data for small RNA sequencing.

Supplementary Table 5 Differentially regulated mRNAs.

Supplementary Table 6 KEGG enrichment of DEGs.

Supplementary Table 7 Detected miRNA.

Supplementary Table 8 Differently expressed miRNAs.

Supplementary Table 9 The primer information of mRNA.
